# 5-Cyclo­hexyl-2-(2-fluoro­phen­yl)-3-methyl­sulfinyl-1-benzo­furan

**DOI:** 10.1107/S1600536813012701

**Published:** 2013-05-15

**Authors:** Hong Dae Choi, Pil Ja Seo, Uk Lee

**Affiliations:** a Department of Chemistry, Dongeui University, San 24 Kaya-dong, Busanjin-gu, Busan 614-714, Republic of Korea; b Department of Chemistry, Pukyong National University, 599-1 Daeyeon 3-dong, Nam-gu, Busan 608-737, Republic of Korea

## Abstract

In the title compound, C_21_H_21_FO_2_S, the cyclo­hexyl ring adopts a chair conformation. The 2-fluoro­benzene ring makes a dihedral angle of 38.68 (6)° with the mean plane [r.m.s. deviation = 0.018 (2) Å] of the benzo­furan fragment. In the crystal, mol­ecules are linked by pairs of C—H⋯O hydrogen bonds into dimers, which are further packed into stacks along the *c* axis by C—H⋯O hydrogen bonds. In addition, the stacked mol­ecules exhibit S⋯O contacts [3.1733 (13) Å] involving the sulfinyl groups. The F atom is disordered over two positions, with site-occupancy factors of 0.961 (3) and 0.039 (3).

## Related literature

For background information and the crystal structures of related compounds, see: Choi *et al.* (2011 ▶, 2012 ▶). For details of sulfin­yl–sulfinyl inter­actions, see: Choi *et al.* (2008 ▶). For a review of carbon­yl–carbonyl inter­actions, see: Allen *et al.* (1998 ▶).
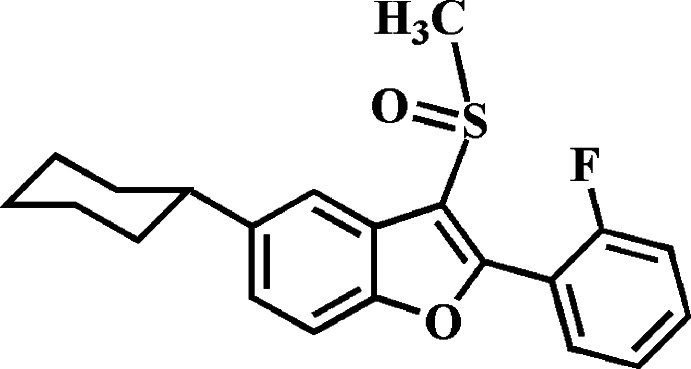


## Experimental

### 

#### Crystal data

C_21_H_21_FO_2_S
*M* *_r_* = 356.44
Monoclinic, 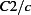

*a* = 33.0231 (12) Å
*b* = 5.6347 (2) Å
*c* = 19.2200 (6) Å
β = 95.855 (2)°
*V* = 3557.7 (2) Å^3^
*Z* = 8
Mo *K*α radiation
μ = 0.20 mm^−1^
*T* = 173 K
0.31 × 0.15 × 0.09 mm

#### Data collection

Bruker SMART APEXII CCD diffractometer
Absorption correction: multi-scan (*SADABS*; Bruker, 2009 ▶) *T*_min_ = 0.545, *T*_max_ = 0.746
29435 measured reflections
4455 independent reflections
3455 reflections with *I* > 2σ(*I*)
*R*_int_ = 0.066

#### Refinement

*R*[*F* ^2^ > 2σ(*F* ^2^)] = 0.045
*wR*(*F* ^2^) = 0.122
*S* = 1.07
4455 reflections
237 parameters
14 restraints
H-atom parameters constrained
Δρ_max_ = 0.36 e Å^−3^
Δρ_min_ = −0.33 e Å^−3^

### 

Data collection: *APEX2* (Bruker, 2009 ▶); cell refinement: *SAINT* (Bruker, 2009 ▶); data reduction: *SAINT*; program(s) used to solve structure: *SHELXS97* (Sheldrick, 2008 ▶); program(s) used to refine structure: *SHELXL97* (Sheldrick, 2008 ▶); molecular graphics: *ORTEP-3 for Windows* (Farrugia, 2012 ▶) and *DIAMOND* (Brandenburg, 1998 ▶); software used to prepare material for publication: *SHELXL97*.

## Supplementary Material

Crystal structure: contains datablock(s) global, I. DOI: 10.1107/S1600536813012701/tk5225sup1.cif

Structure factors: contains datablock(s) I. DOI: 10.1107/S1600536813012701/tk5225Isup2.hkl

Supplementary material file. DOI: 10.1107/S1600536813012701/tk5225Isup3.cml

Additional supplementary materials:  crystallographic information; 3D view; checkCIF report

## Figures and Tables

**Table 1:** Hydrogen-bond geometry (Å, °)

*D*—H⋯*A*	*D*—H	H⋯*A*	*D*⋯*A*	*D*—H⋯*A*
C19—H19⋯O2^i^	0.95	2.44	3.220 (2)	140
C21—H21*A*⋯O2^ii^	0.98	2.57	3.265 (2)	128

Symmetry codes: (i) 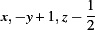; (ii) 
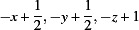
.

**Fig. 1.**
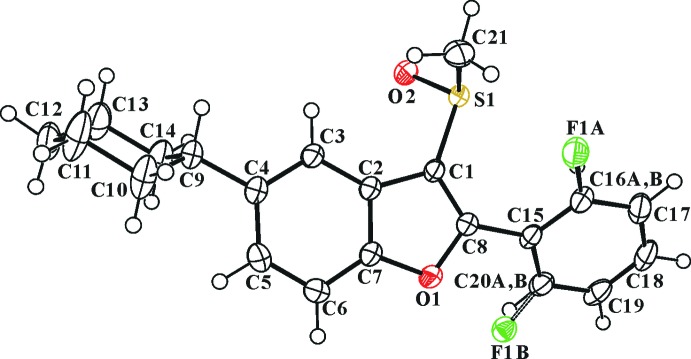
The molecular structure of the title compound with the atom numbering scheme. Displacement ellipsoids are drawn at the 50% probability level. H atoms are presented as small spheres of arbitrary radius. The F atom of the 2-fluorophenyl ring is disordered over two positions with site occupancy factors, from refinement of 0.961 (3) (part A) and 0.039 (3) (part B).

**Fig. 2.**
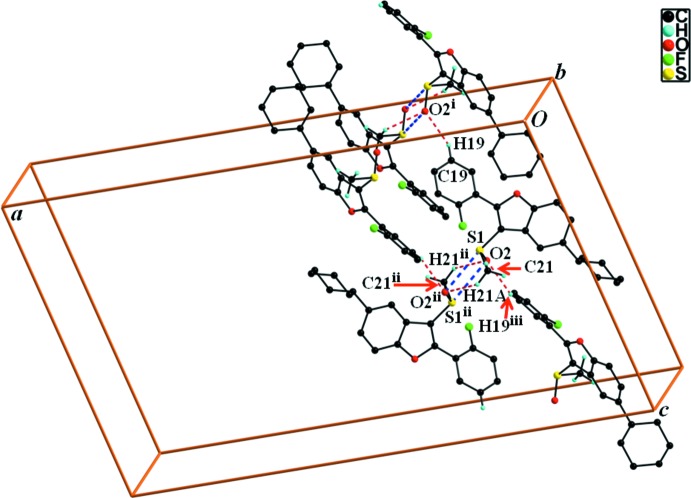
A view of the C—H···O and S···O interactions (dotted lines) in the crystal structure of the title compound. H atoms non-participating in hydrogen-bonding and disordered part B atoms were omitted for clarity. [Symmetry codes: (i) xX, - y + 1, z - 1/2; (ii) - x + 1/2, - y + 1/2, - z + 1; (iii) x, - y + 1, z + 1/2 .]

## supplementary crystallographic information

### Comment

As a part of our continuing study of 5-cyclohexyl-3-methylsulfinyl-1-benzofuran derivatives containing 4-fluorophenyl (Choi *et al.*, 2011) and 3-fluorophenyl (Choi *et al.*, 2012) substituents in 2-position, we report herein the crystal structure of the title compound.

In the title molecule (Fig. 1), the benzofuran unit is essentially planar, with a mean deviation of 0.018 (2) Å from the least-squares plane defined by the nine constituent atoms. In the 2-fluorobenzene ring, the F atom is disordered over two positions with site-occupancy factors, from refinement, of 0.961 (3) (part A) and 0.039 (3) (part B). The cyclohexyl ring has the chair form. The dihedral angle formed by the 2-fluorobenzene ring and the mean plane of the benzofuran fragment is 38.68 (6)°. In the crystal structure (Fig. 2), molecules are linked by pairs of C—H···O hydrogen bonds into centrosymmetric dimers, which are further packed into stacks along the *c* axis by C—H···O hydrogen bonds (Table 1). In addition, the crystal packing (Fig. 2) exhibits a sulfinyl–sulfinyl interaction (Choi *et al.*, 2008) similar to a type-II carbonyl-carbonyl interaction (Allen *et al.*, 1998), with a S1···O2^ii^ distance of 3.1733 (13) Å (symmetry operation *ii*: 1/2-*x*, 1/2-*y*, 1-*z*).

### Experimental

3-Chloroperoxybenzoic acid (77%, 208 mg, 0.9 mmol) was added in small portions to a stirred solution of 5-cyclohexyl-2-(2-fluorophenyl)-3-methylsulfanyl-1-benzofuran (271 mg, 0.8 mmol) in dichloromethane (30 mL) at 273 K. After being stirred at room temperature for 5 h, the mixture was washed with saturated sodium bicarbonate solution and the organic layer was separated, dried over magnesium sulfate, filtered and concentrated at reduced pressure. The residue was purified by column chromatography (hexane–ethyl acetate, 2:1 v/v) to afford the title compound as a colorless solid [yield 78%, M.pt: 452–453 K; *R*_f_ = 0.59 (hexane–ethyl acetate, 2:1 v/v)]. Single crystals suitable for X-ray diffraction were prepared by slow evaporation of a solution of the title compound in ethyl acetate at room temperature.

### Refinement

All H atoms were positioned geometrically and refined using a riding model, with C—H = 0.95 Å for aryl, 1.00 Å for methine, 0.99 Å for methylene and 0.98Å for methyl H atoms, respectively, and with *U*_iso_(H) = 1.2*U*_eq_(C) for aryl, methine and methylene, and 1.5*U*_eq_(C) for methyl H atoms. The positions of methyl hydrogens were optimized rotationally. The F1 atom of the 2-fluorophenyl ring is disordered over two positions with site occupancy factors, from refinement, of 0.961 (3) (part A) and 0.039 (3) (part B). For the proper treatment of H-atoms, carbon atoms C16 and C20 were divided in two parts with equalized coordinates and thermal parameters. The distance of equivalent C—F pairs were restrained to 1.330 (5) Å using command DFIX, and displacement ellipsoids of F1 set were restrained to 0.01 using command ISOR.

### Crystal data

**Table:**

C_21_H_21_FO_2_S	*F*(000) = 1504
*M**_r_* = 356.44	*D*_x_ = 1.331 Mg m^−^^3^
Monoclinic, *C*2/*c*	Mo *K*α radiation, λ = 0.71073 Å
Hall symbol: -C 2yc	Cell parameters from 7608 reflections
*a* = 33.0231 (12) Å	θ = 2.4–27.9°
*b* = 5.6347 (2) Å	µ = 0.20 mm^−^^1^
*c* = 19.2200 (6) Å	*T* = 173 K
β = 95.855 (2)°	Block, colourless
*V* = 3557.7 (2) Å^3^	0.31 × 0.15 × 0.09 mm
*Z* = 8	

### Data collection

**Table:**

Bruker SMART APEXII CCD diffractometer	4455 independent reflections
Radiation source: rotating anode	3455 reflections with *I* > 2σ(*I*)
Graphite multilayer monochromator	*R*_int_ = 0.066
Detector resolution: 10.0 pixels mm^-1^	θ_max_ = 28.4°, θ_min_ = 2.1°
φ and ω scans	*h* = −44→42
Absorption correction: multi-scan (*SADABS*; Bruker, 2009)	*k* = −5→7
*T*_min_ = 0.545, *T*_max_ = 0.746	*l* = −25→25
29435 measured reflections	

### Refinement

**Table:**

Refinement on *F*^2^	Primary atom site location: structure-invariant direct methods
Least-squares matrix: full	Secondary atom site location: difference Fourier map
*R*[*F*^2^ > 2σ(*F*^2^)] = 0.045	Hydrogen site location: difference Fourier map
*wR*(*F*^2^) = 0.122	H-atom parameters constrained
*S* = 1.07	*w* = 1/[σ^2^(*F*_o_^2^) + (0.0503*P*)^2^ + 2.7846*P*] where *P* = (*F*_o_^2^ + 2*F*_c_^2^)/3
4455 reflections	(Δ/σ)_max_ = 0.001
237 parameters	Δρ_max_ = 0.36 e Å^−^^3^
14 restraints	Δρ_min_ = −0.33 e Å^−^^3^

### Special details

**Table:**

Geometry. All esds (except the esd in the dihedral angle between two l.s. planes) are estimated using the full covariance matrix. The cell esds are taken into account individually in the estimation of esds in distances, angles and torsion angles; correlations between esds in cell parameters are only used when they are defined by crystal symmetry. An approximate (isotropic) treatment of cell esds is used for estimating esds involving l.s. planes.
Refinement. Refinement of F^2^ against ALL reflections. The weighted R-factor wR and goodness of fit S are based on F^2^, conventional R-factors R are based on F, with F set to zero for negative F^2^. The threshold expression of F^2^ > 2sigma(F^2^) is used only for calculating R-factors(gt) etc. and is not relevant to the choice of reflections for refinement. R-factors based on F^2^ are statistically about twice as large as those based on F, and R- factors based on ALL data will be even larger.

### Fractional atomic coordinates and isotropic or equivalent isotropic displacement parameters (Å^2^)

**Table:**

	*x*	*y*	*z*	*U*_iso_*/*U*_eq_	Occ. (<1)
S1	0.206366 (12)	0.27237 (7)	0.42614 (2)	0.02355 (12)	
O1	0.11688 (4)	0.5340 (3)	0.29615 (6)	0.0347 (3)	
O2	0.21653 (4)	0.4453 (2)	0.48408 (6)	0.0320 (3)	
C1	0.16017 (5)	0.3784 (3)	0.38215 (8)	0.0233 (3)	
C2	0.12801 (5)	0.4947 (3)	0.41447 (9)	0.0251 (4)	
C3	0.11880 (5)	0.5338 (3)	0.48284 (9)	0.0271 (4)	
H3	0.1356	0.4687	0.5212	0.033*	
C4	0.08520 (6)	0.6673 (4)	0.49437 (9)	0.0340 (4)	
C5	0.06078 (7)	0.7595 (4)	0.43703 (11)	0.0494 (6)	
H5	0.0377	0.8519	0.4454	0.059*	
C6	0.06884 (7)	0.7218 (4)	0.36850 (11)	0.0497 (6)	
H6	0.0519	0.7849	0.3300	0.060*	
C7	0.10277 (5)	0.5879 (4)	0.35935 (9)	0.0330 (4)	
C8	0.15236 (5)	0.4084 (3)	0.31233 (8)	0.0252 (4)	
C9	0.07601 (6)	0.7166 (4)	0.56872 (10)	0.0358 (4)	
H9	0.0931	0.6047	0.5996	0.043*	
C10	0.03215 (7)	0.6704 (5)	0.58103 (12)	0.0508 (6)	
H10A	0.0251	0.5041	0.5685	0.061*	
H10B	0.0141	0.7755	0.5504	0.061*	
C11	0.02494 (8)	0.7145 (5)	0.65731 (13)	0.0620 (8)	
H11A	−0.0042	0.6906	0.6631	0.074*	
H11B	0.0409	0.5990	0.6877	0.074*	
C12	0.03733 (7)	0.9641 (5)	0.67931 (12)	0.0505 (6)	
H12A	0.0194	1.0792	0.6522	0.061*	
H12B	0.0338	0.9858	0.7295	0.061*	
C13	0.08082 (7)	1.0129 (5)	0.66758 (11)	0.0504 (6)	
H13A	0.0990	0.9109	0.6990	0.061*	
H13B	0.0874	1.1804	0.6794	0.061*	
C14	0.08855 (7)	0.9658 (4)	0.59173 (11)	0.0465 (5)	
H14A	0.0731	1.0822	0.5609	0.056*	
H14B	0.1179	0.9881	0.5868	0.056*	
C15	0.17462 (5)	0.3439 (3)	0.25303 (8)	0.0263 (4)	
C16A	0.19517 (6)	0.1306 (3)	0.25044 (9)	0.0301 (4)	0.961 (3)
F1A	0.19125 (4)	−0.0328 (2)	0.30066 (6)	0.0405 (4)	0.961 (3)
C16B	0.19517 (6)	0.1306 (3)	0.25044 (9)	0.0301 (4)	0.04
H16B	0.1930	0.0181	0.2867	0.036*	0.039 (3)
C17	0.21859 (6)	0.0748 (4)	0.19744 (10)	0.0370 (5)	
H17	0.2329	−0.0714	0.1979	0.044*	
C18	0.22086 (6)	0.2346 (4)	0.14361 (10)	0.0383 (5)	
H18	0.2371	0.1992	0.1068	0.046*	
C19	0.19974 (6)	0.4449 (4)	0.14305 (9)	0.0374 (5)	
H19	0.2010	0.5530	0.1054	0.045*	
C20A	0.17669 (6)	0.4997 (4)	0.19703 (9)	0.0317 (4)	0.961 (3)
H20A	0.1621	0.6452	0.1960	0.038*	0.961 (3)
C20B	0.17669 (6)	0.4997 (4)	0.19703 (9)	0.0317 (4)	0.04
F1B	0.1515 (6)	0.680 (3)	0.1832 (13)	0.031 (7)	0.039 (3)
C21	0.18645 (6)	0.0135 (3)	0.46404 (10)	0.0360 (4)	
H21A	0.2084	−0.0696	0.4923	0.054*	
H21B	0.1746	−0.0918	0.4268	0.054*	
H21C	0.1654	0.0599	0.4938	0.054*	

### Atomic displacement parameters (Å^2^)

**Table:**

	*U*^11^	*U*^22^	*U*^33^	*U*^12^	*U*^13^	*U*^23^
S1	0.0256 (2)	0.0259 (2)	0.0196 (2)	0.00267 (16)	0.00441 (15)	0.00009 (16)
O1	0.0323 (7)	0.0526 (8)	0.0196 (6)	0.0133 (6)	0.0049 (5)	0.0022 (6)
O2	0.0346 (7)	0.0348 (7)	0.0257 (6)	0.0027 (5)	−0.0020 (5)	−0.0065 (5)
C1	0.0271 (8)	0.0246 (8)	0.0186 (8)	0.0012 (7)	0.0045 (6)	−0.0007 (6)
C2	0.0257 (8)	0.0292 (9)	0.0209 (8)	0.0016 (7)	0.0054 (6)	0.0005 (7)
C3	0.0263 (8)	0.0339 (9)	0.0215 (8)	0.0018 (7)	0.0046 (7)	0.0008 (7)
C4	0.0352 (10)	0.0427 (11)	0.0254 (9)	0.0066 (8)	0.0096 (8)	−0.0003 (8)
C5	0.0427 (12)	0.0707 (16)	0.0366 (11)	0.0285 (11)	0.0126 (9)	0.0036 (11)
C6	0.0448 (12)	0.0762 (16)	0.0283 (10)	0.0309 (11)	0.0047 (9)	0.0060 (10)
C7	0.0325 (10)	0.0469 (11)	0.0203 (9)	0.0098 (8)	0.0063 (7)	0.0005 (8)
C8	0.0259 (8)	0.0285 (9)	0.0214 (8)	0.0027 (7)	0.0033 (6)	−0.0007 (7)
C9	0.0378 (10)	0.0461 (11)	0.0253 (9)	0.0097 (9)	0.0110 (8)	−0.0015 (8)
C10	0.0497 (13)	0.0640 (15)	0.0423 (12)	−0.0144 (11)	0.0225 (10)	−0.0213 (11)
C11	0.0598 (15)	0.0855 (19)	0.0462 (13)	−0.0247 (14)	0.0324 (12)	−0.0241 (13)
C12	0.0426 (12)	0.0723 (16)	0.0386 (12)	0.0047 (11)	0.0134 (10)	−0.0210 (11)
C13	0.0461 (12)	0.0668 (16)	0.0399 (12)	−0.0060 (11)	0.0113 (10)	−0.0195 (11)
C14	0.0444 (12)	0.0585 (14)	0.0390 (12)	−0.0084 (10)	0.0163 (9)	−0.0109 (10)
C15	0.0283 (9)	0.0329 (9)	0.0180 (8)	−0.0016 (7)	0.0037 (6)	−0.0032 (7)
C16A	0.0364 (10)	0.0315 (10)	0.0227 (9)	−0.0017 (8)	0.0044 (7)	−0.0041 (7)
F1A	0.0590 (8)	0.0315 (6)	0.0326 (7)	0.0058 (5)	0.0117 (5)	0.0008 (5)
C16B	0.0364 (10)	0.0315 (10)	0.0227 (9)	−0.0017 (8)	0.0044 (7)	−0.0041 (7)
C17	0.0361 (10)	0.0422 (11)	0.0336 (10)	0.0015 (8)	0.0071 (8)	−0.0124 (9)
C18	0.0362 (10)	0.0545 (13)	0.0255 (9)	−0.0047 (9)	0.0096 (8)	−0.0142 (9)
C19	0.0396 (11)	0.0545 (13)	0.0187 (9)	−0.0068 (9)	0.0059 (8)	0.0012 (8)
C20A	0.0335 (10)	0.0391 (11)	0.0223 (9)	0.0002 (8)	0.0025 (7)	0.0007 (8)
C20B	0.0335 (10)	0.0391 (11)	0.0223 (9)	0.0002 (8)	0.0025 (7)	0.0007 (8)
F1B	0.030 (10)	0.036 (11)	0.028 (10)	−0.002 (8)	0.006 (7)	0.000 (8)
C21	0.0427 (11)	0.0311 (10)	0.0350 (10)	0.0002 (8)	0.0070 (9)	0.0103 (8)

### Geometric parameters (Å, º)

**Table:**

S1—O2	1.4921 (13)	C11—C12	1.513 (3)
S1—C1	1.7708 (17)	C11—H11A	0.9900
S1—C21	1.7856 (19)	C11—H11B	0.9900
S1—O2^i^	3.1733 (13)	C12—C13	1.502 (3)
O1—C8	1.377 (2)	C12—H12A	0.9900
O1—C7	1.379 (2)	C12—H12B	0.9900
C1—C8	1.351 (2)	C13—C14	1.529 (3)
C1—C2	1.442 (2)	C13—H13A	0.9900
C2—C7	1.383 (2)	C13—H13B	0.9900
C2—C3	1.396 (2)	C14—H14A	0.9900
C3—C4	1.377 (2)	C14—H14B	0.9900
C3—H3	0.9500	C15—C16A	1.384 (3)
C4—C5	1.398 (3)	C15—C20A	1.396 (2)
C4—C9	1.516 (2)	C16A—F1A	1.3495 (19)
C5—C6	1.387 (3)	C16A—C17	1.377 (2)
C5—H5	0.9500	C17—C18	1.380 (3)
C6—C7	1.377 (3)	C17—H17	0.9500
C6—H6	0.9500	C18—C19	1.374 (3)
C8—C15	1.464 (2)	C18—H18	0.9500
C9—C10	1.514 (3)	C19—C20A	1.383 (3)
C9—C14	1.517 (3)	C19—H19	0.9500
C9—H9	1.0000	C20A—H20A	0.9500
C10—C11	1.529 (3)	C21—H21A	0.9800
C10—H10A	0.9900	C21—H21B	0.9800
C10—H10B	0.9900	C21—H21C	0.9800
			
O2—S1—C1	104.66 (7)	C12—C11—H11B	109.5
O2—S1—C21	107.05 (9)	C10—C11—H11B	109.5
C1—S1—C21	97.77 (9)	H11A—C11—H11B	108.1
O2—S1—O2^i^	74.92 (6)	C13—C12—C11	111.33 (19)
C1—S1—O2^i^	173.96 (6)	C13—C12—H12A	109.4
C21—S1—O2^i^	76.76 (7)	C11—C12—H12A	109.4
C8—O1—C7	105.70 (13)	C13—C12—H12B	109.4
C8—C1—C2	107.27 (15)	C11—C12—H12B	109.4
C8—C1—S1	125.75 (13)	H12A—C12—H12B	108.0
C2—C1—S1	125.75 (12)	C12—C13—C14	111.50 (18)
C7—C2—C3	119.27 (15)	C12—C13—H13A	109.3
C7—C2—C1	104.79 (14)	C14—C13—H13A	109.3
C3—C2—C1	135.90 (16)	C12—C13—H13B	109.3
C4—C3—C2	119.62 (16)	C14—C13—H13B	109.3
C4—C3—H3	120.2	H13A—C13—H13B	108.0
C2—C3—H3	120.2	C9—C14—C13	111.81 (19)
C3—C4—C5	119.06 (17)	C9—C14—H14A	109.3
C3—C4—C9	119.58 (17)	C13—C14—H14A	109.3
C5—C4—C9	121.34 (17)	C9—C14—H14B	109.3
C6—C5—C4	122.73 (18)	C13—C14—H14B	109.3
C6—C5—H5	118.6	H14A—C14—H14B	107.9
C4—C5—H5	118.6	C16A—C15—C20A	117.12 (15)
C7—C6—C5	116.29 (19)	C16A—C15—C8	122.04 (16)
C7—C6—H6	121.9	C20A—C15—C8	120.82 (16)
C5—C6—H6	121.9	F1A—C16A—C17	118.39 (17)
C6—C7—O1	125.86 (17)	F1A—C16A—C15	118.93 (15)
C6—C7—C2	123.02 (17)	C17—C16A—C15	122.65 (17)
O1—C7—C2	111.09 (15)	C16A—C17—C18	118.91 (19)
C1—C8—O1	111.12 (14)	C16A—C17—H17	120.5
C1—C8—C15	133.03 (16)	C18—C17—H17	120.5
O1—C8—C15	115.78 (14)	C19—C18—C17	120.16 (17)
C10—C9—C4	113.72 (17)	C19—C18—H18	119.9
C10—C9—C14	110.52 (17)	C17—C18—H18	119.9
C4—C9—C14	111.67 (17)	C18—C19—C20A	120.30 (18)
C10—C9—H9	106.8	C18—C19—H19	119.8
C4—C9—H9	106.8	C20A—C19—H19	119.8
C14—C9—H9	106.8	C19—C20A—C15	120.78 (18)
C9—C10—C11	111.53 (18)	C19—C20A—H20A	119.6
C9—C10—H10A	109.3	C15—C20A—H20A	119.6
C11—C10—H10A	109.3	S1—C21—H21A	109.5
C9—C10—H10B	109.3	S1—C21—H21B	109.5
C11—C10—H10B	109.3	H21A—C21—H21B	109.5
H10A—C10—H10B	108.0	S1—C21—H21C	109.5
C12—C11—C10	110.8 (2)	H21A—C21—H21C	109.5
C12—C11—H11A	109.5	H21B—C21—H21C	109.5
C10—C11—H11A	109.5		
			
O2—S1—C1—C8	131.23 (16)	C7—O1—C8—C15	−176.16 (16)
C21—S1—C1—C8	−118.80 (17)	C3—C4—C9—C10	−132.0 (2)
O2—S1—C1—C2	−34.53 (17)	C5—C4—C9—C10	49.6 (3)
C21—S1—C1—C2	75.45 (16)	C3—C4—C9—C14	102.1 (2)
C8—C1—C2—C7	−0.1 (2)	C5—C4—C9—C14	−76.3 (3)
S1—C1—C2—C7	167.87 (14)	C4—C9—C10—C11	178.0 (2)
C8—C1—C2—C3	−178.0 (2)	C14—C9—C10—C11	−55.5 (3)
S1—C1—C2—C3	−10.0 (3)	C9—C10—C11—C12	56.3 (3)
C7—C2—C3—C4	−1.0 (3)	C10—C11—C12—C13	−55.9 (3)
C1—C2—C3—C4	176.7 (2)	C11—C12—C13—C14	55.2 (3)
C2—C3—C4—C5	0.5 (3)	C10—C9—C14—C13	54.5 (3)
C2—C3—C4—C9	−177.98 (17)	C4—C9—C14—C13	−177.83 (17)
C3—C4—C5—C6	0.2 (4)	C12—C13—C14—C9	−54.7 (3)
C9—C4—C5—C6	178.6 (2)	C1—C8—C15—C16A	39.3 (3)
C4—C5—C6—C7	−0.3 (4)	O1—C8—C15—C16A	−143.89 (17)
C5—C6—C7—O1	−178.3 (2)	C1—C8—C15—C20A	−139.2 (2)
C5—C6—C7—C2	−0.2 (4)	O1—C8—C15—C20A	37.6 (2)
C8—O1—C7—C6	176.9 (2)	C20A—C15—C16A—F1A	−174.72 (16)
C8—O1—C7—C2	−1.4 (2)	C8—C15—C16A—F1A	6.7 (3)
C3—C2—C7—C6	0.9 (3)	C20A—C15—C16A—C17	3.3 (3)
C1—C2—C7—C6	−177.5 (2)	C8—C15—C16A—C17	−175.27 (17)
C3—C2—C7—O1	179.23 (16)	F1A—C16A—C17—C18	176.19 (17)
C1—C2—C7—O1	0.9 (2)	C15—C16A—C17—C18	−1.9 (3)
C2—C1—C8—O1	−0.8 (2)	C16A—C17—C18—C19	−0.5 (3)
S1—C1—C8—O1	−168.73 (13)	C17—C18—C19—C20A	1.2 (3)
C2—C1—C8—C15	176.11 (19)	C18—C19—C20A—C15	0.4 (3)
S1—C1—C8—C15	8.2 (3)	C16A—C15—C20A—C19	−2.5 (3)
C7—O1—C8—C1	1.3 (2)	C8—C15—C20A—C19	176.08 (17)

Symmetry code: (i) −*x*+1/2, −*y*+1/2, −*z*+1.

### Hydrogen-bond geometry (Å, º)

**Table:**

*D*—H···*A*	*D*—H	H···*A*	*D*···*A*	*D*—H···*A*
C19—H19···O2^ii^	0.95	2.44	3.220 (2)	140
C21—H21*A*···O2^i^	0.98	2.57	3.265 (2)	128

Symmetry codes: (i) −*x*+1/2, −*y*+1/2, −*z*+1; (ii) *x*, −*y*+1, *z*−1/2.
